# Population-Level Surveillance of Domestic Assaults in the Home Using the National Emergency Medical Services Information System (NEMSIS)

**DOI:** 10.1007/s11121-024-01683-w

**Published:** 2024-05-30

**Authors:** Millan Alexander AbiNader, Andrew G. Rundle, Yoosun Park, Alexander X. Lo

**Affiliations:** 1 The School of Social Policy and Practice, The University of Pennsylvania, Philadelphia, PA USA; 2 Mailman School of Public Health, Columbia University, New York, NY USA; 3 Dept. of Emergency Medicine, Center for Health Services Outcomes Research, Northwestern University Feinberg School of Medicine, Northwestern University, Chicago, IL USA

**Keywords:** Domestic violence, NEMSIS, Emergency medical services, Surveillance

## Abstract

Violence in the home, including partner violence, child abuse, and elder abuse, is pervasive in the United States. An informatics approach allowing automated analysis of administrative data to identify domestic assaults and release timely and localized data would assist preventionists to identify geographic and demographic populations of need and design tailored interventions. This study examines the use of an established national dataset, the NEMSIS 2019, as a potential annual automated data source for domestic assault surveillance. An algorithm was used to identify individuals who utilized emergency medical services (EMS) for a physical assault in a private residence (*N* = 176,931). Descriptive analyses were conducted to define the identified population and disposition of patients. A logistic regression was performed to predict which characteristics were associated with consistent domestic assault identification by the on-scene EMS clinician and dispatcher. The sample was majority female (52.2%), White (44.7%), urban (85.5%), and 21–29 years old (24.4%). A disproportionate number of those found dead on scene were men (74.5%), and female patients more often refused treatment (57.8%) or were treated and then released against medical advice (58.4%). Domestic assaults against children and seniors had higher odds of being consistently identified by both the dispatcher and EMS clinician than those 21–49, and women had lower odds of consistent identification than men. While a more specific field to identify the type of domestic assault (e.g., intimate partner) would help inform specialized intervention planning, these data indicate an opportunity to systematically track domestic assaults in communities and describe population-specific needs.

**Supplementary Information:**

The online version contains supplementary material available at 10.1007/s11121-024-01683-w.

## Introduction

Domestic assault, or violent assaults occurring between members of the same household, family members or intimate partners, occurs throughout the life course and across communities in the U.S. In the U.S., 60% of simple and aggravated assaults occur in private residences in 2021 (FBI, 2022). Nearly half of adult women (41%), one fourth of adult men, and one third of transgender adults experience intimate partner violence (IPV) in their lifetimes (Langenderfer-Magruder et al., 2016; Leemis et al., 2022). One in 7 children experiences child abuse and neglect and 1 in 10 seniors experiences abuse in the home (CDC, 2021, 2022). IPV (Campbell, 2002), child abuse and neglect (Norman et al., 2012), and elder abuse (Yunus et al., 2019) have been linked to numerous negative individual-level health outcomes, and the burden of violence extends beyond the home. IPV’s estimated lifetime cost in the U.S. is $4.7 trillion, 59% of which are medical costs and 37% of which are paid for using public funds (Peterson et al., 2018). The total annual U.S. cost of child abuse and neglect is estimated at $161 billion, and lifetime medical expenses average around $52,000 per child (Fang et al., 2012). While the cost of physical violence against seniors has not yet been estimated, financial elder abuse alone is estimated to cost $3.4 billion per year (MetLife Mature Market Institute, 2013).

Communities responding to the burden of domestic assaults require localized data about the distribution, severity, and cost of domestic assault to develop effective interventions and identify populations that have the most need. Two nationally representative datasets are available to estimate victimization in the U.S.: the National Intimate Partner and Sexual Violence Survey (NISVS) and the National Survey of Victims of Crime (NCVS). The NISVS is a Centers for Disease Control and Prevention surveillance system that surveys non-institutionalized U.S. adults about lifetime and past year intimate partner and sexual violence victimization and its health impacts using random-digit dialing (Kresnow et al., 2022). The NISVS began collecting data in 2010 and captures an in-depth picture of victimization types, perpetrator-victim relationships, and the impacts of violence and report estimates at the national level (Kresnow et al., 2022). The Bureau of Justice Statistics manages the NCVS surveillance system, an annual survey of U.S. household members ages 12 and older that started in 1973. Participants are interviewed about their experience with a range of crimes and their use of the criminal justice system (Bureau of Justice Statistics, 2021). While both national datasets offer an opportunity to examine domestic assaults and help-seeking behavior after victimization throughout the life course, the data are unavailable at a local level, and there is a time lag in their release, limiting their utility to inform current local intervention needs for policy makers and systems that respond to violence in the home.

Local domestic assault estimates predominantly rely on samples drawn from criminal justice, medical, and social services settings. Local police data, for example, are available through the Federal Bureau of Investigation’s National Incident-Based Reporting System (NIBRS), allowing public users to access local estimates through an online surveillance portal (AbiNader et al., 2023; FBI, 2022). While these data sources may accurately capture victims with a help-seeking propensity, it is likely that they fail to capture the total prevalence of domestic assaults. Less than half of women experiencing IPV report it to the police (44%) (Holliday et al., 2020), and calling the police has been linked to more injurious IPV (Ogbonnaya et al., 2021). Examining racial differences, Holliday and colleagues (2020) found that Black women had higher rates of reporting than White women and that Latina and White women had similar rates of reporting. However, Black and Latina women were more likely to report IPV to law enforcement if it resulted in injury (Holliday et al., 2020). Similarly, those experiencing more injurious violence are more likely to seek emergency medical care (Duterte et al., 2008), limiting the completeness of emergency room data. Moreover, previous studies suggest that only about 14% of assault victims access emergency medical services (Kaylen & Pridemore, 2015). Child and adult protective service samples overrepresent children and adult victims of color, generating data samples with potential racial bias (Drake et al., 2011; Lachs et al., 1997). Community-based domestic violence services serve predominantly cis-heterosexual women, thereby underestimating the prevalence among LGBTQIA individuals and men (Lyon et al., 2008). In rural communities, barriers to accessing criminal justice, medical, and social services such as transportation may also result in a lack of rural representation in datasets (Grama, 2000; Peek-Asa et al., 2011). Despite these limitations, service population data can offer insight into the demographics of individuals using local services, the types and context of violence experienced, the type and efficacy of interventions, and the relative spatial distribution of need and services. Understanding the burden of disease at the local level is imperative to allow policy makers to allocate funding and interventions to the most needed areas and to track the impacts of those interventions.

An informatics approach to understanding the health burden of domestic assaults that allows automated analysis of administrative data to release more timely and localized data would support prevention and response resources. A data-driven public health surveillance approach has long been called for as a necessary tool to track and understand the prevalence and etiology of injury and the effectiveness of interventions (Thacker & Berkelman, 1988). Public health surveillance requires that data are translated for individuals that design policies and programs in order to promptly respond to changes in the burden and context of a disease (Thacker & Berkelman, 1988). Surveillance must be ongoing and timely in order to actively and efficiently respond to problems (Jajosky & Groseclose, 2004; Thacker & Berkelman, 1988). Moreover, this type of systematic data collection could also track how victims move through systems and the effectiveness of interventions. This latter information can be used for routine system evaluation to constantly improve responses to victims of violence.

Here, using data from the National Emergency Medical Services Information System (NEMSIS), we estimate the number of identified incidents of domestic assaults that occurred in personal residences and required an emergency medical service (EMS) response in the U.S. We describe the epidemiologic characteristics of these complaints in terms of the patient’s age, sex, race, and ethnicity, by month of the year, day of the week and by time of day, and by census region. In doing so, we create an algorithm to identify EMS responses to incidents of domestic assaults using NEMSIS data. We describe the strengths and limitations of this novel approach with the expectation that analytical methods can be developed for routine use with local or regional EMS administrative data for public health surveillance of domestic assaults.

## Methods

### Study Design and Data Source

We conducted a retrospective analysis using publicly available 2019 NEMSIS research data. NEMSIS is a product of the National Highway Traffic Safety Administration Office of Emergency Medical Services and is the largest repository of de-identified EMS records in the U.S., with over 34 million events from more than 10,000 EMS agencies (Dawson, 2006; Hanlin et al., 2022). For this study we used the most recent NEMSIS dataset (2019 version) available (https://nemsis.org/using-ems-data/request-research-data/) prior to the COVID-19 pandemic, in order to avoid the well-documented impact of the COVID-19 pandemic and stay-at-home orders on many aspects of community interaction, arguably affecting domestic assaults in particular (Kourti et al., 2023) and on EMS response patterns that potentially affected responses to domestic assault calls (Al Amiry & Maguire, 2021; Handberry et al., 2021). We believe the 2019 NEMSIS data most accurately reflects the true patterns of domestic assault exclusive of the influence of COVID-19. To our knowledge, this is the first use of NEMSIS data to describe and measure domestic assaults. NEMSIS data are released as a de-identified, Health Insurance Portability and Accountability Act exempt, publicly available dataset, hosted by the University of Utah with local Institutional Review Board (IRB) oversight (https://nemsis.org/using-ems-data/). Therefore, no further IRB review was sought for these analyses.

### Inclusion of Records

Records were excluded if the Disposition (eDisposition.12) of the response was listed as Canceled (codes 4,212,007, 4,212,009, 4,212,011), Standby-No Services or Support Provided (code 4,212,039), or Transport Non-patient (code 4,212,043) or eResponse.05 was coded as Interfacility Transport (code 2,205,003) or Medical Transport (code 2,205,007).

### Measures

EMS activations for which the dispatcher reported domestic assaults were identified if the NEMSIS eDispatch.01 variable was listed as Assault or Stab/Gunshot Wound/Penetrating trauma and the NEMSIS eScene.09 variable was listed as Private Residence. To code patients as having experienced domestic assaults based on clinical data reported by the EMS clinical on scene, ICD10 coded data from the eInjury.01, eSituation.11 and eSituation.12 variables were used. The eInjury.01 variable records the cause of treated injuries, and the eSituation variables record the EMS clinician’s overall impression of the encounter. Patients treated in private residences were coded as having experienced domestic assault if the eSituation variables reported Neglect or Abuse or the eInjury.01 variable included codes for Neglect, Abuse, or Assault.

The eDisposition.12 variable indicated the patient’s final disposition, such as treated on scene, transported to hospital, or died on scene. Patient acuity is categorized by EMS clinicians using nationally standardized definitions for “Critical,” “Emergent,” and “Lower Acuity,” based on the Patient Acuity Definitions defined by the NHTSA National EMS Core Content and which follows the Model of Clinical Practice of Emergency Medicine (National Highway Traffic Safety Administration, 2005). The NEMSIS eSituation.13 variable reports the responding EMS clinicians’ rating of the patient’s condition on scene. The eSituation.13 variable adds a fourth category “Dead without Resuscitation Efforts” to these three acuity categories. The eSituation.13 variable has missing values, particularly when the eDisposition.12 variable was coded as “Patient evaluated, no treatment or transport required” or “Patient treated and released per protocol.” Thus, when eSituation.13 had missing values and eDsiposition.12 was coded as “Patient evaluated, no treatment or transport required” or “Patient treated and released per protocol,” the patient was coded as Lower Acuity for the purposes of analyses.

### Sociodemographic Data

NEMSIS variables were used to categorize the age (ageyears), sex (ePatient.13), and race and ethnicity (ePatient.14) of the patient. The CensusDivision variable was used to define the U.S. region where the incident occurred.

### Statistical Analyses

Patients were coded as having experienced domestic assault based on EMS dispatch data and based on the EMS clinicians’ on-scene evaluation. Descriptive statistics were used to describe patient sociodemographic characteristics and acuity based on EMS clinician data. Sensitivity analyses were run on the sociodemographic and patient acuity data to compare results when EMS clinician data were used to define domestic assaults and when EMS dispatch data were used to define domestic assaults. No substantive differences were found in magnitude nor direction (Online Resource 1).

Cross tabulations were used to assess the agreement between the EMS dispatch data and the EMS clinician for classifying patients as having experienced domestic assault. EMS dispatchers receive information from a variety of people with varying levels of knowledge about an incident, and dispatch data reflect the content of what they are told. Thus, EMS dispatch reports are highly variable, dependent upon the quality of information provided to the dispatcher, and with a significant frequency of discordance with the true clinical situation on scene (Bohm & Kurland, 2018; Reckdenwald et al., 2017). By contrast, the on-scene EMS clinician is able to assess the true context of the emergency and connect with emergency department clinicians to discuss any abuse situations. Therefore, it is likely that the EMS clinical report more accurately represents the incident than the dispatcher report. The sensitivity, specificity, and positive predictive value for the dispatch data predicting the EMS clinician report data were calculated. Logistic regression analyses were used to determine whether patient characteristics and time of day were associated with the EMS clinician evaluation agreeing with the dispatch information. For all EMS activations that were classified as domestic assaults based on the dispatch data, activations were coded as Agree if the EMS clinician data also identified a domestic assault and were coded as Disagree if the EMS clinician data did not identify a domestic assault. Among patients classified as having experienced domestic assault using the EMS clinician data, descriptive analyses were conducted for the patient acuity, final disposition of the EMS response, and patient socio-demographics.

## Results

In total 12,426,669 EMS activations were dispatched to private residences in 2019. Based on dispatch data, 218,595 EMS activations were classified as being for domestic assaults, while EMS clinician data identified 176,931 patients who had experienced domestic assaults. The sensitivity and specificity for the dispatch data predicting that the EMS clinician data would identify domestic assaults were 62% and 99%, respectively, and the positive predictive value was 51%.

Table 1 reports on the analyses of predictors of the EMS clinician identification of domestic assaults agreeing with the dispatch data. Compared to the patient age being < 21 years, agreement was positively associated with the patient’s age being above 65 or not reported and negatively associated with age less than 50. Female sex being reported for the patient was associated with a lower odds of agreement, and sex not being reported was associated with higher odds of the two data sources agreeing. Patient race and ethnicity were not associated with the two data sources agreeing. The odds of agreement between the data sources were substantially higher for EMS dispatches occurring between 9 a.m. and 7 p.m., as compared to dispatches occurring between midnight and 1 a.m.

**Table 1:** Predictors of whether the EMS clinician data agree with the dispatch data for a domestic assault^a^

Parameter	OR	95% Wald confidence interval for OR	
		Lower CI	Upper CI
Age of patient			
< 21	1		
21–29	0.870	0.842	0.900
30–39	0.859	0.830	0.889
40–49	0.908	0.875	0.943
50–64	1.020	0.983	1.058
65+	2.258	2.149	2.373
Not recorded	7.769	6.837	8.828
Sex of patient			
Male	1		
Female	0.880	0.861	0.899
Not recorded	5.015	4.481	5.613
Race and ethnicity of patient			
American Indian or Alaska Native	1		
Asian	1.148	0.973	1.354
Black or African American	1.048	0.920	1.194
Hispanic or Latin	1.077	0.940	1.233
Native Hawaiian or Other Pacific Islander	1.205	0.936	1.550
White	1.080	0.950	1.229
Mixed race	1.095	0.883	1.358
Not recorded	1.092	0.959	1.242
Time of day			
0:00–0:59	1		
1:00–1:59	1.033	0.968	1.103
2:00–2:59	0.973	0.908	1.043
3:00–3:59	1.014	0.943	1.090
4:00–4:59	1.083	0.999	1.173
5:00–5:59	1.100	1.008	1.199
6:00–6:59	1.153	1.052	1.262
7:00–7:59	1.187	1.089	1.295
8:00–8:59	1.073	0.988	1.166
9:00–9:59	1.217	1.125	1.316
10:00–10:59	1.198	1.110	1.292
11:00–11:59	1.195	1.110	1.287
12:00–12:59	1.181	1.099	1.268
13:00–13:59	1.219	1.136	1.308
14:00–14:59	1.153	1.075	1.237
15:00–15:59	1.161	1.085	1.242
16:00–16:59	1.173	1.099	1.252
17:00–17:59	1.125	1.056	1.200
18:00–18:59	1.113	1.046	1.185
19:00–19:59	1.037	0.975	1.102
20:00–20:59	1.044	0.983	1.109
21:00–21:59	1.040	0.980	1.104
22:00–22:59	0.991	0.934	1.053
23:00–23:59	0.984	0.926	1.046
(Intercept)	0.794	0.692	0.911

^a^Among all patients coded as having experienced domestic assault using the dispatch data

Table 2 reports on the sociodemographic characteristics of the patients classified as having experienced domestic assaults by the EMS clinician. EMS activations for domestic assaults were highest during the summer. Overall, the patients were majority White, urban, 21–29 years old, and from the South Atlantic region and were relatively balanced across the sexes: 47% were male, 52% were female, and 0.57% did not have a sex listed in the data. Female patients were more likely to be in the age range of 21 to 39 years of age. The distribution of patients by race and ethnicity, Census Division, urbanicity, and month of the year of the incident did not vary by sex.

**Table 2:** Sociodemographiccharacteristics of patients classified as having experienced domestic assaults using the EMS clinician data

	Total sample		Sex of patient					
			Male		Female		Not recorded	
	Count	Column *N*%	Count	Column *N*%	Count	Column *N*%	Count	Column *N*%
Total *N*	176,931	100.0%	83,515		92,403		1013	100.0%
Age group								
< 21	29,592	16.7%	14,354	17.2%	15,097	16.3%	141	13.9%
21–29	43,094	24.4%	18,400	22.0%	24,472	26.5%	222	21.9%
30–39	39,901	22.6%	17,507	21.0%	22,193	24.0%	201	19.8%
40–49	26,105	14.8%	12,378	14.8%	13,622	14.7%	105	10.4%
50–64	27,818	15.7%	15,607	18.7%	12,091	13.1%	120	11.8%
65+	9590	5.4%	4882	5.8%	4665	5.0%	43	4.2%
Not recorded	831	0.5%	387	0.5%	263	0.3%	181	17.9%
Race and ethnicity								
American Indian or Alaska Native	840	0.7%	408	0.7%	427	0.7%	5	0.7%
Asian	1183	0.9%	562	1.0%	614	0.9%	7	0.9%
Black or African American	20,176	16.2%	9614	16.3%	10,417	16.0%	145	19.7%
Hispanic or Latin	6820	5.5%	3212	5.4%	3579	5.5%	29	3.9%
Native Hawaiian or Other Pacific Islander	261	0.2%	129	0.2%	129	0.2%	3	0.4%
White	55,864	44.7%	26,379	44.6%	29,182	44.9%	303	41.1%
Mixed Race	443	0.4%	229	0.4%	210	0.3%	4	0.5%
Not recorded	39,322	31.5%	18,575	31.4%	20,506	31.5%	241	32.7%
Census Division								
East North Central	17,656	10.0%	8336	10.0%	9242	10.0%	78	7.7%
East South Central	11,441	6.5%	5350	6.4%	5882	6.4%	209	20.6%
Middle Atlantic	9271	5.2%	4365	5.2%	4893	5.3%	13	1.3%
Mountain	21,863	12.4%	9998	12.0%	11,753	12.7%	112	11.1%
New England	4990	2.8%	2336	2.8%	2639	2.9%	15	1.5%
Pacific	24,924	14.1%	12,666	15.2%	12,131	13.1%	127	12.5%
South Atlantic	46,649	26.4%	22,145	26.5%	24,257	26.3%	247	24.4%
West North Central	8762	5.0%	4126	4.9%	4586	5.0%	50	4.9%
West South Central	31,296	17.7%	14,144	16.9%	16,990	18.4%	162	16.0%
Not recorded	79	0.0%	49	0.1%	30	0.0%	0	0.0%
Urbanicity								
Urban	151,227	85.5%	71,174	85.2%	79,159	85.7%	894	88.3%
Suburban	8117	4.6%	3924	4.7%	4175	4.5%	18	1.8%
Rural	10,203	5.8%	4866	5.8%	5269	5.7%	68	6.7%
Wilderness	2661	1.5%	1286	1.5%	1362	1.5%	13	1.3%
Not recorded	4723	2.7%	2265	2.7%	2438	2.6%	20	2.0%
Month								
January	13,257	7.5%	6269	7.5%	6909	7.5%	79	7.8%
February	11,900	6.7%	5598	6.7%	6239	6.8%	63	6.2%
March	13,827	7.8%	6554	7.8%	7188	7.8%	85	8.4%
April	14,742	8.3%	6863	8.2%	7805	8.4%	74	7.3%
May	16,359	9.2%	7789	9.3%	8478	9.2%	92	9.1%
June	16,515	9.3%	7913	9.5%	8508	9.2%	94	9.3%
July	16,800	9.5%	7957	9.5%	8739	9.5%	104	10.3%
August	16,492	9.3%	7856	9.4%	8552	9.3%	84	8.3%
September	15,437	8.7%	7313	8.8%	8051	8.7%	73	7.2%
October	15,143	8.6%	7120	8.5%	7919	8.6%	104	10.3%
November	13,174	7.4%	6227	7.5%	6871	7.4%	76	7.5%
December	13,285	7.5%	6056	7.3%	7144	7.7%	85	8.4%

Table 3 reports on the patient acuity and the final disposition of the patient. In total, patient acuity data was missing from 21.7% of patients: 22.0% for male and 21.1% for female patients. But among those with data, a greater percentage of male patients had acuity grades of Emergent, Critical, and Found dead without resuscitation efforts than female patients. The final disposition of male patients was more likely to be “Patient died at scene” and “Patient treated and transferred or transported” than for female patients. Female patients were more likely to be listed as “Patient evaluated no treatment or transport required,” “Patient refused evaluation,” “Patient treated and released AMA,” or “Patient treated and released per protocol” than male patients.

**Table 3:** Acuity and disposition of the patients classified as having experienced domestic assault using the EMS clinician data

	Total *N*		Sex of patient					
			Male		Female		Not recorded	
	Count	Column *N*%	Count	Column *N*%	Count	Column *N*%	Count	Column *N*%
Total *N*	176,931	100.0%	83,515	100.0%	92,403	100.0%	1013	100.0%
Patient acuity at time of EMS response								
Lower Acuity^a^	111,446	63.0%	49,066	58.8%	61,919	67.0%	461	45.5%
Emergent	21,600	12.2%	12,076	14.5%	9435	10.2%	89	8.8%
Critical	4886	2.8%	3532	4.2%	1325	1.4%	29	2.9%
Dead without resuscitation efforts	668	0.4%	480	0.6%	182	0.2%	6	0.6%
Not reported	38,331	21.7%	18,361	22.0%	19,542	21.1%	428	42.3%
Disposition of response								
Patient died at scene	1116	0.6%	831	1.0%	277	0.3%	8	0.8%
Patient evaluated, no treatment or transport required	12,293	6.9%	4928	5.9%	7263	7.9%	102	10.1%
Patient refused evaluation	19,821	11.2%	8215	9.8%	11,465	12.4%	141	13.9%
Patient treated and released AMA	27,322	15.4%	11,274	13.5%	15,949	17.3%	99	9.8%
Patient treated and released per protocol	7976	4.5%	3299	4.0%	4595	5.0%	82	8.1%
Patient treated and transferred or transported	107,017	60.5%	54,283	65.0%	52,336	56.6%	398	39.3%
Other	1386	0.8%	685	0.8%	518	0.6%	183	18.1%

^a^When eSituation.13 had missing values and eDsiposition.12 was coded as “Patient evaluated, no treatment or transport required” or “Patient treated and released per protocol,” the patient was coded as Lower Acuity for analyses

## Discussion

This paper used a novel approach to demonstrate how administrative data may be leveraged to develop local, automated surveillance systems to track domestic assaults. The results suggest that data provided by the dispatch record is not a sufficiently sensitive screen for identifying patients who had experienced domestic assault. Instead, data reported by the EMS clinician in the eInjury and eSituation variables should be used to identify patients who have experienced domestic assault. Analyses of the eInjury and eSituation variables are more challenging than analyses of the eDispatch variable but can be automated and offer a promising new approach to surveillance of domestic assaults.

The NEMSIS data mirrors trends in national law enforcement data on assaults. The NIBRS similarly reports that the largest age group experiencing simple and aggravated assaults are in their 20s, and more White individuals experience assault (FBI, 2022). Like the NEMSIS data, NIBRS estimates that more men die of fatal assaults than women (FBI, 2022). Recent NCVS data report that urban areas experience higher rates of assault incidents than suburban and rural areas, similar to the NEMSIS service-use estimates (Thompson et al., 2022). These parallel findings suggest that NEMSIS may be an excellent system to leverage to produce local estimates of domestic assaults.

Women were less likely to be consistently identified as domestic assault victims in the EMS data. More research is needed to determine if the definitions of domestic assault are not accurately capturing women’s experiences of violence in the home or if this points to women being more likely to recant an account of violence due to safety concerns or to systemic barriers (Bennett et al., 1999; Epstein & Goodman, 2018). More work is needed to determine if the finding that women more frequently refuse care is related to a lack of gender-responsive care, gendered perceptions of criminal legal systems, or due to gendered differences in the types of assault experienced, as women are more likely to experience simple assault than men (FBI, 2022).

Findings from the NEMSIS data indicate an opportunity to systematically track domestic assaults in communities and describe population-specific needs. The predictive validity analyses suggest that while the dispatch data is good at predicting non-domestic assault cases (specificity, 99%), its identification of domestic assaults could be improved (sensitivity, 62%; positive predictive value, 51%). Although the findings from the regression model suggest that dispatch data may be good at capturing elder abuse and abuse towards children and youth, it appears less good at capturing violence in middle age. In sum, these analyses suggest that any automated surveillance system would need to use the more complex EMS clinician data than the simpler dispatch data. Future implementation studies may also examine and test trainings on domestic assault for dispatchers to improve accurate reporting (Reckdenwald et al., 2017). As occurs in many administrative datasets, the missingness in the race/ethnicity and acuity data presents challenges for identifying groups at increased risk of violence and informing interventions that are appropriate for intersectional identities. While this causes limitations in public health surveillance, this approach would still likely improve upon current methods. Moreover, should NEMSIS be formally expanded into a surveillance tool, its expanded use in this way may encourage less missing data in years to come.

The primary strength of NEMSIS data is that they provide a well-documented census of health complaints requiring an EMS response across 47 U.S. states. NEMSIS has established standardized variables for which data need to be reported by all EMS, and these required variables were used to create our domestic assault case–finding algorithm. The domestic assault case–identification strategy relies on ICD 10 codes entered into these specific data fields by EMS responders. Much of the data is supplied to NEMSIS by third-party companies that provide data services to EMS companies and health care systems. The data entry systems provided by these third-party companies vary, and this may influence the ICD 10 coding options available to EMS personnel; thus, the quality of the ICD 10 data may vary by provider and between regions these companies operate in. However, these data service companies could begin selling “public health surveillance as service” data products by city and region and develop a sustainable model for routine analyses. In addition, if NEMSIS had the capacity to institute Data Use Agreements with county health departments that allowed access to data with county identifiers, NEMSIS EMS data could be routinely analyzed locally for local public health surveillance purposes.

A limitation of the informatics approach demonstrated here is that the coding schema do not make use of narratives and text notes created by EMS personnel. As such, the reliance on ICD 10 codes may result in an undercount of incidents of domestic assaults, particularly those that occurred outside the home or between partners or family members that do not currently cohabitate. However, machine learning for natural language processing applied to EMS narrative notes could supplement the ICD 10-based case-finding algorithm and increase the sensitivity of identifying incidents of domestic assaults from EMS data, as has been used elsewhere with fatal assault data (Kafka et al., 2023). Further research is needed to determine the feasibility of automated processing of EMS notes to identify domestic assaults and whether increased sensitivity comes at the cost of reduced specificity. Despite these limitations, the NEMSIS data demonstrate a unique opportunity to expand an existing data system to better track domestic assaults locally.

## Conclusion

NEMSIS data represent an opportunity to estimate the health burden of domestic assaults in communities. The approach to analyzing administrative data described here provides the foundation for an automated, routine public health surveillance of domestic assaults, particularly those resulting in injury. Such surveillance systems would support the planning and allocation of resources for treatment and social and mental health services and would support targeted community-level and institutional prevention intervention strategies.

## Supplementary Information

Below is the link to the electronic supplementary material.

Supplementary file1 (DOCX 36 KB)

## Data Availability

EMS data are available by request at: https://nemsis.org/
