# Supplementary material for: Population-Level Surveillance of Domestic Assaults in the Home Using the National Emergency Medical Services Information System (NEMSIS)
Source: Prev Sci. 2024 May 30;25(6):882–90. doi: 10.1007/s11121-024-01683-w (PMC11390926; doi:10.1007/s11121-024-01683-w)
Supplement: Supplementary file 1 — Supplementary file1 (DOCX 36 KB) [file 11121_2024_1683_MOESM1_ESM.docx]

**Online Resource, Sensitivity Analysis: Table 2**

*Sociodemographic Characteristics of Patients Classified as Having Experienced Domestic Assaults using the EMS Dispatch Data*

|  | Total Sample | | Sex of patient | | | | | |
| --- | --- | --- | --- | --- | --- | --- | --- | --- |
|  |  |  | Male | | Female | | Not Recorded | |
|  | Count | Column N % | Count | Column N % | Count | Column N % | Count | Column N % |
| Total N | 218595 | 100.0% | 97590 | 100.0% | 106883 | 100.0% | 14122 | 100.0% |
| Age Group |  |  |  |  |  |  |  |  |
| <21 | 34927 | 16.0% | 16807 | 17.2% | 17722 | 16.6% | 398 | 2.8% |
| 21 - 29 | 49766 | 22.8% | 21275 | 21.8% | 28006 | 26.2% | 485 | 3.4% |
| 30-39 | 45135 | 20.6% | 19837 | 20.3% | 24829 | 23.2% | 469 | 3.3% |
| 40-49 | 29549 | 13.5% | 13922 | 14.3% | 15376 | 14.4% | 251 | 1.8% |
| 50-64 | 31934 | 14.6% | 17623 | 18.1% | 14018 | 13.1% | 293 | 2.1% |
| 65+ | 13560 | 6.2% | 7176 | 7.4% | 6218 | 5.8% | 166 | 1.2% |
| Not Recorded | 13724 | 6.3% | 950 | 1.0% | 714 | 0.7% | 12060 | 85.4% |
| Race and Ethnicity |  |  |  |  |  |  |  |  |
| American Indian or Alaska Native | 1024 | 0.7% | 481 | 0.7% | 476 | 0.6% | 67 | 0.6% |
| Asian | 1519 | 1.0% | 678 | 1.0% | 748 | 1.0% | 93 | 0.9% |
| Black or African American | 24559 | 16.0% | 10956 | 16.0% | 11882 | 15.9% | 1721 | 16.5% |
| Hispanic or Latin | 8397 | 5.5% | 3721 | 5.4% | 4105 | 5.5% | 571 | 5.5% |
| Native Hawaiian or Other Pacific Islander | 350 | 0.2% | 177 | 0.3% | 150 | 0.2% | 23 | 0.2% |
| White | 68910 | 44.8% | 30641 | 44.7% | 33654 | 44.9% | 4615 | 44.2% |
| Mixed Race | 573 | 0.4% | 263 | 0.4% | 258 | 0.3% | 52 | 0.5% |
| Not Recorded | 48508 | 31.5% | 21603 | 31.5% | 23597 | 31.5% | 3308 | 31.7% |
| Census Division |  |  |  |  |  |  |  |  |
| East North Central | 19502 | 8.9% | 8971 | 9.2% | 10348 | 9.7% | 183 | 1.3% |
| East South Central | 13094 | 6.0% | 6106 | 6.3% | 6698 | 6.3% | 290 | 2.1% |
| Middle Atlantic | 15276 | 7.0% | 7059 | 7.2% | 7887 | 7.4% | 330 | 2.3% |
| Mountain | 25019 | 11.4% | 11449 | 11.7% | 12743 | 11.9% | 827 | 5.9% |
| New England | 5674 | 2.6% | 2628 | 2.7% | 2954 | 2.8% | 92 | 0.7% |
| Pacific | 25382 | 11.6% | 12206 | 12.5% | 11642 | 10.9% | 1534 | 10.9% |
| South Atlantic | 61904 | 28.3% | 27221 | 27.9% | 29077 | 27.2% | 5606 | 39.7% |
| West North Central | 12430 | 5.7% | 5051 | 5.2% | 5322 | 5.0% | 2057 | 14.6% |
| West South Central | 40209 | 18.4% | 16832 | 17.2% | 20176 | 18.9% | 3201 | 22.7% |
| Not Recorded | 19502 | 8.9% | 8971 | 9.2% | 10348 | 9.7% | 183 | 1.3% |
| Urbanicity |  |  |  |  |  |  |  |  |
| Urban | 183136 | 83.8% | 80711 | 82.7% | 88997 | 83.3% | 13428 | 95.1% |
| Suburban | 10762 | 4.9% | 5178 | 5.3% | 5401 | 5.1% | 183 | 1.3% |
| Rural | 14138 | 6.5% | 6625 | 6.8% | 7233 | 6.8% | 280 | 2.0% |
| Wilderness | 3622 | 1.7% | 1733 | 1.8% | 1835 | 1.7% | 54 | 0.4% |
| Not Recorded | 6937 | 3.2% | 3343 | 3.4% | 3417 | 3.2% | 177 | 1.3% |
| Month |  |  |  |  |  |  |  |  |
| January | 16659 | 7.6% | 7466 | 7.7% | 8120 | 7.6% | 1073 | 7.6% |
| February | 14933 | 6.8% | 6700 | 6.9% | 7261 | 6.8% | 972 | 6.9% |
| March | 16934 | 7.7% | 7570 | 7.8% | 8355 | 7.8% | 1009 | 7.1% |
| April | 17848 | 8.2% | 8022 | 8.2% | 8758 | 8.2% | 1068 | 7.6% |
| May | 19842 | 9.1% | 8831 | 9.0% | 9701 | 9.1% | 1310 | 9.3% |
| June | 19884 | 9.1% | 9025 | 9.2% | 9720 | 9.1% | 1139 | 8.1% |
| July | 20761 | 9.5% | 9261 | 9.5% | 9977 | 9.3% | 1523 | 10.8% |
| August | 20262 | 9.3% | 9134 | 9.4% | 9778 | 9.1% | 1350 | 9.6% |
| September | 19022 | 8.7% | 8452 | 8.7% | 9348 | 8.7% | 1222 | 8.7% |
| October | 18792 | 8.6% | 8242 | 8.4% | 9251 | 8.7% | 1299 | 9.2% |
| November | 16622 | 7.6% | 7502 | 7.7% | 8067 | 7.5% | 1053 | 7.5% |
| December | 17036 | 7.8% | 7385 | 7.6% | 8547 | 8.0% | 1104 | 7.8% |

**Online Resource, Sensitivity Analysis: Table 3**

*Acuity and Disposition of the Patients Classified as Having Experienced Domestic Assault using the EMS Dispatch Data*

|  | Total N | | Sex of patient | | | | | |
| --- | --- | --- | --- | --- | --- | --- | --- | --- |
|  |  |  | Male | | Female | | Not Recorded | |
|  | Count | Column N % | Count | Column N % | Count | Column N % | Count | Column N % |
| Total N | 218595 | 100.0% | 97590 | 100.0% | 106883 | 100.0% | 14122 | 100.0% |
| Patient acuity at time of EMS response |  |  |  |  |  |  |  |  |
| Lower Acuity | 120255 | 55.0% | 50935 | 52.2% | 67681 | 63.3% | 1639 | 11.6% |
| Emergent | 24184 | 11.1% | 13613 | 13.9% | 10361 | 9.7% | 210 | 1.5% |
| Critical | 8417 | 3.9% | 5982 | 6.1% | 1984 | 1.9% | 451 | 3.2% |
| Dead without Resuscitation Efforts | 2641 | 1.2% | 2100 | 2.2% | 494 | 0.5% | 47 | 0.3% |
| Not Reported | 63098 | 28.9% | 24960 | 25.6% | 26363 | 24.7% | 11775 | 83.4% |
| Disposition of Response |  |  |  |  |  |  |  |  |
| Patient died at scene | 4319 | 2.0% | 3482 | 3.6% | 768 | 0.7% | 69 | 0.5% |
| Patient evaluated, no treatment or transport required | 16385 | 7.5% | 6154 | 6.3% | 9595 | 9.0% | 636 | 4.5% |
| Patient refused evaluation | 31660 | 14.5% | 11491 | 11.8% | 17221 | 16.1% | 2948 | 20.9% |
| Patient treated and released AMA | 28387 | 13.0% | 11311 | 11.6% | 16947 | 15.9% | 129 | 0.9% |
| Patient treated and released per protocol | 10087 | 4.6% | 3911 | 4.0% | 5987 | 5.6% | 189 | 1.3% |
| Patient treated and transferred or transported | 114563 | 52.4% | 59306 | 60.8% | 54621 | 51.1% | 636 | 4.5% |
| Other | 13194 | 6.0% | 1935 | 2.0% | 1744 | 1.6% | 9515 | 67.4% |
